# Pulmonary Function and Persistent Clinical Symptoms in Children and Their Parents 12 Months After Mild SARS-CoV-2 Infection

**DOI:** 10.3389/fped.2022.894331

**Published:** 2022-06-30

**Authors:** Sebastian F. N. Bode, Marisa Haendly, Dorit Fabricius, Benjamin Mayer, Maria Zernickel, Anneke Donne Maree Haddad, Pauline Frieh, Roland Elling, Hanna Renk, Maximilian Stich, Eva-Maria Jacobsen, Klaus-Michael Debatin, Ales Janda

**Affiliations:** ^1^Department of Pediatrics and Adolescent Medicine, Ulm University Medical Center, Ulm University, Ulm, Germany; ^2^Institute of Epidemiology and Medical Biometry, Ulm University, Ulm, Germany; ^3^Center for Pediatrics and Adolescent Medicine, Medical Center Freiburg, Germany and Faculty of Medicine, University of Freiburg, Freiburg, Germany; ^4^Institute for Immunodeficiency, Medical Center Freiburg, Germany and Faculty of Medicine, University of Freiburg, Freiburg, Germany; ^5^University Children’s Hospital Tübingen, Tübingen, Germany; ^6^Department of Pediatrics, University Children’s Hospital Heidelberg, Heidelberg, Germany

**Keywords:** spirometry, children, adolescents, SARS-CoV-2, COVID-19, convalescence

## Abstract

**Background:**

Pulmonary involvement is the leading cause of morbidity and mortality after severe acute respiratory syndrome coronavirus-2 (SARS-CoV-2) infection. Long-term impairment has been reported in adults with severe infection. However, most infections cause only mild symptoms or are even asymptomatic, especially in children. There is insufficient evidence regarding pulmonary outcome measures in mild SARS-CoV-2. The objectives of this study were to determine spirometry parameters after SARS-CoV-2 infection and correlate those with reported persisting symptoms in children, adolescents, and adults.

**Methods:**

Data on clinical symptoms during acute infection as well as SARS-CoV-2 serology results were recorded. Twelve months after infection, spirometry was performed and information on persisting symptoms was collected using a structured questionnaire. 182 participants (108 SARS-CoV-2 positive) from 48 families were included; 53 children (< 14 years), 34 adolescents and young adults (14–25 years), and 95 adults.

**Results:**

Spirometry values did not significantly differ between the particular subgroups of the cohort (adults, adolescents, children; infected and non-infected individuals). Adults reported more symptoms during acute infection as well more persisting fatigue (29.7% of participants), reduced physical resilience (34.4%), and dyspnea (25.0%) 12 months after infection than adolescents (fatigue 26.7%, reduced physical resilience 20%, and 0% dyspnea) and children (4%, 0%, 0%, respectively). There was no correlation between persistent subjective symptoms and spirometry results.

**Discussion:**

Children and adolescents are less affected than adults by acute SARS-CoV-2 as well as by post-infection persistent symptoms. Spirometry was not able to demonstrate any differences between healthy individuals and participants who had suffered from mild SARS-CoV-2 12 months after the infection.

## Introduction

Since the end of 2019, severe acute respiratory syndrome coronavirus 2 (SARS-CoV-2) has spread globally and caused more than 370 million infections worldwide to date. Over 5.6 million deaths have been reported due to the new coronavirus disease (COVID-19) in this global pandemic [COVID-19 dashboard^1^, (1)]. Morbidity and mortality have significantly impacted health care providers and communities. However, most infected individuals show a mild clinical course and many are asymptomatic (2, 3). Children typically have less severe disease and are more often asymptomatic than adults (4–6).

Pulmonary involvement is the major factor for morbidity and mortality in COVID-19 (7, 8). Acute pulmonary involvement is well documented; hypoxemia and need for non-invasive ventilation, mechanical ventilation or even extracorporeal membrane oxygenation are common (8–10). Typical radiological changes include ground-glass opacities with or without consolidations, pleural or interlobal thickening and bronchoaerograms (11). Pathological changes are manifold and can resemble acute respiratory distress syndrome (ARDS), bacterial or viral pneumonia/pneumonitis, vasculitis with (micro-) thrombosis, amyloid deposition, or organizing pneumonia (12–15).

Persistent dyspnea and cough have been reported even months after hospital discharge (16, 17). Adult survivors of severe COVID-19 show restrictive and, less frequently, obstructive changes in lung function tests (18). A reduced carbon-monoxide (CO) diffusion capacity as well as a reduced 6-min walking distance, and oxygen desaturation on exercise have been reported 4 months after COVID-19 (19). Even in mild cases without hospitalization, persistent pulmonary symptoms, such as dyspnea and reduced exercise capacity, and reduced pulmonary function test results have been reported 2 months after infection (20).

Little is known about the differences in pulmonary function tests and subjective persistent symptoms in children, adolescents and adults more than just a few months after mild SARS-CoV-2 infection. We prospectively assessed households with SARS-CoV-2 infected and non-infected parents and their children and performed spirometry around 12 months after infection. We correlated infection status and spirometry parameters with reported acute symptoms during infection and persistent chronic symptoms.

## Materials and Methods

### Study Design

We conducted a single-center, cross-sectional study to assess pulmonary function after mild SARS-CoV-2 infection in children (< 14 years), adolescents (14–25 years), and adults (> 25 years), and correlated results with SARS-CoV-2 serology and reported symptoms. This study is part of the COVID-19 BaWü prospective household study, where families with at least one SARS-CoV-2 PCR-confirmed or seropositive index case of SARS-CoV-2 were included. Cohort data on intrafamilial transmission dynamics of SARS-CoV-2 (21) and long term serologic responses after SARS-CoV-2 infection (22) have been published. Participants were recruited and first examined (SARS-CoV-2 serology, clinical data collection) between April and August 2020 (T1) at Freiburg, Tübingen, Heidelberg and Ulm University Medical Centers in Baden-Württemberg, Germany. Second visit (T2) with blood drawing took place between February and May 2021. Voluntary recruitment of participants took place during the first wave of the SARS-CoV-2 pandemic between May and August 2020 *via* local health authorities and by the means of in-hospital databases of families with at least one laboratory-confirmed SARS-CoV-2 infection. Only participants recruited at Ulm University were subjected to spirometry measurement at T2 (approx. 12 months after SARS-CoV-2 infection, the time-point was set by the part of the study focusing on serology) and are part of this study. Participants or their parents gave written consent to participate in the study.

### Spirometry

Spirometry was performed with the Easy On-PC spirometer (ndd Medical Technologies, Andover, MA, United States) after [23] standards and was evaluated with the EasyOne Connect platform (ndd Medical Technologies, Andover, MA, United States). Z-scores for forced vital capacity (FVC), forced expiratory volume in 1 s (FEV1), FVC/FEV1, forced expiratory flow (FEF) at 75% of FVC (FEF 75%) as well as FEF25–75 were recorded (24) and are reported as FVCz, FEV1z, FVC/FEV1z, FEF75z, and FEF25–75z, respectively. Individuals who were not able to perform spirometry of adequate quality according to ATS/ERS guidelines were not included in the study, for example children < 4 years as they are not able to perform spirometry maneuvers sufficiently. Participants were grouped according to their age as children [age 4–14 years, adolescents/young adults ≥ 14–25 years, and adults (i.e., the parents of the participating children/adolescents)].

### Serology and Clinical Data

SARS-CoV-2 seropositivity at T1 (approx. 4 months after infection with the virus) was used as a proxy of previous SARS-CoV-2 infection. Serological response was measured using EuroImmun-Anti-SARS-CoV-2 ELISA for anti-Spike-1 IgG, Siemens Healthineers SARS-CoV-2 for anti-RBD (receptor-binding domain) IgG and Roche Elecsys for anti-NCP (nucleocapsid-protein) IgM and IgG. Seropositivity was defined as at least any two of the three SARS-CoV-2 assays being positive [Details in Renk et al. (22)]. In the subcohort that performed spirometry no participants were hospitalized, therefore all SARS-CoV-2 infections were classified as mild or asymptomatic, depending on reported clinical data.

At T1 a questionnaire comprising demographical data, and clinical symptoms as fever, cough, diarrhea, and dysgeusia around the time of SARS-CoV-2 infection was filled out by participants or their parents (for children < 14 years of age). At T2, participants completed an online questionnaire [*via* REDCap©^2^ (25, 26)] that addressed a range of symptoms and health outcomes. In the present study, we focus on questions regarding three self-perceived subjective symptoms relevant to pulmonary involvement (fatigue, reduced physical resilience and dyspnea), their perceived severity and duration, and the extent to which they limited daily activities, all in comparison before the SARS-CoV-2 infection. Persistent symptoms were classified as symptoms still present at T2. The cohort reported here is a sub-cohort of a larger, multi-center cohort and post-/long-COVID symptoms are reported elsewhere for the whole cohort (27). Smoking status and pre-existing pulmonary conditions were recorded.

### Statistics

Statistical analysis was performed with SAS^®^ Studio 3.4 (SAS^®^, Cary, NC, United States). Figures were prepared using Graph Pad Prism (Version 7.01, GraphPad Software, La Jolla, CA, United States^3^). Frequencies for categorical data and medians as well as interquartile ranges (IQR) are reported for metric variables. Non-parametric Spearman rank correlation was calculated and interpreted according to Cohen (28). Wilcoxon rank sum tests were used to determine differences in medians of lung function parameters. An explorative type 1 error level was set as *p* < 0.05.

### Ethics

Ethics approval was obtained from the Ulm University ethics committee (permit no: 152/20). The study was conducted according to the Declaration of Helsinki. It was designed, analyzed and reported according to the Strengthening the Reporting of Observational Studies in Epidemiology (STROBE) guidelines. The study was registered in the German registry for clinical trials (DRKS registry, study ID 00021521).

## Results

Totally 182 participants (53 children 4–14 years, 34 adolescents 14–25 years, 95 adults > 25 years; 89 females, 93 males) performed spirometry (Table 1) and were included into the study. 108 participants (59.3%) had positive SARS-CoV-2 serology at T1 and were thus deemed as having had a SARS-CoV-2 infection. Adults were significantly more often seropositive (65.3%) as well as symptomatic (90.7% of seropositive adults) during acute infection than were adolescents (47.1% seropositive and 37.5% of seropositive adolescents were symptomatic) and children (52.8% seropositive and 67.9% of seropositive children were symptomatic) (Table 1).

**TABLE 1:** Demographics and key serologic and clinical information on the study participants.

	Children (< 14 y-o)	Adolescents (14–25 y-o)	Adults (> 25 y-o)
N (% of total)	53 (29.1)	34 (18.7)	95 (52.2)
Age [y]: median (IQR)	9.4 (3.9)	16.2 (3.2)	46.4 (7.8)
Number of females (%)	25 (47.2)	16 (47.1)	48 (50.5)
BMI [kg/m^2^]: median (IQR)	16.6 (3.6)	21.7 (3.8)	26.4 (5.6)
Number of SARS-CoV-2 seropositive participants (% of age group)	28 (52.8)	16 (47.1)	62 (65.3)
Number of asymptomatic participants (% of seropositive)	9 (32.1)	10 (62.5)	6 (9.7)
**Symptoms at disease onset (% of seropositive participants)**			
Fever (%)	10 (35.7)	5 (31.3)	32 (51.6)
Cough (%)	8 (28.6)	2 (12.5)	31 (50.0)
Dysgeusia (%)	1 (3.6)	3 (18.8)	30 (48.4)
Diarrhea (%)	2 (7.1)	0 (0)	9 (14.5)
Median (IQR) days from positive PCR test result (or symptom onset) to spirometry measurement (T2)	348 (29.4)	360 (16.5)	364 (13)
Smoker [n]	0	3	2
Asthma [n]	0	0	4
Pollen allergy [n]	0	1	3

*IQR, interquartile range; N, number.*

*Smokers defined as current smoker or ex-smoker that quit < 10 years ago; y-o: years of age.*

### Spirometry

**FIGURE 1:**
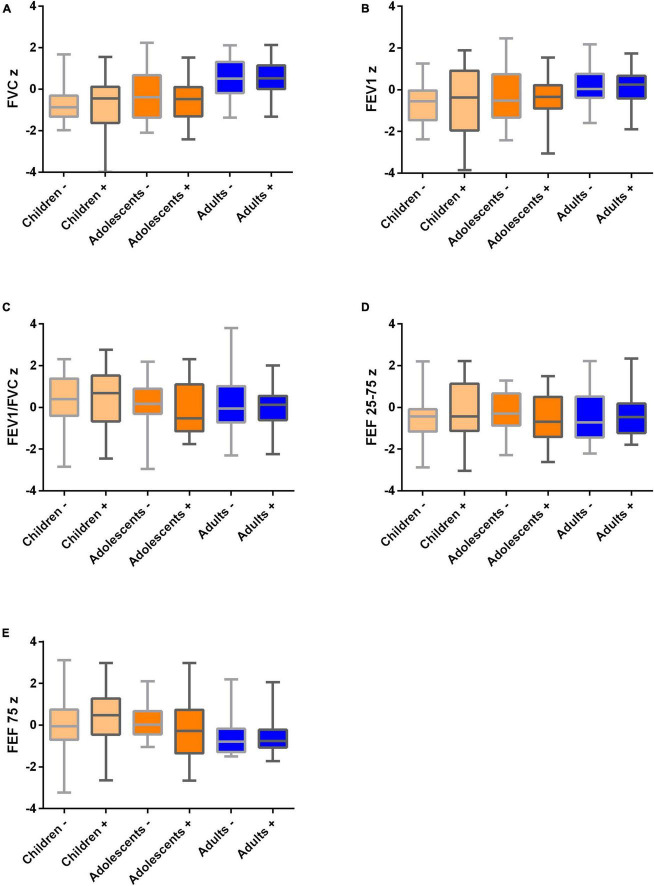
Spirometry values in children (*n* = 53), adolescents and young adults (*n* = 34) and their parents (*n* = 95) 12 months after mild SARS-CoV-2 infection in *n* = 25/15/68 participants, respectively, and healthy family members as controls. SARS-CoV-2 negative (-) vs. positive (+) participants are visualized. All medians are in the normal range. Boxplots (medians, quartiles, minimum and maximum values) for normalized (z scores) spirometry parameters. **(A)** FVCz, **(B)** FEV1z, **(C)** FEV1z/FVCz, **(D)** FEF 25–75z, and **(E)** FEF 75z. FVC, forced vital capacity; FEV1, forced expiratory volume in 1 s; FEF25 75, mean flow between 25% and 75% of the forced vital capacity; FEF75, maximum expiratory flow at 75% expiration of forced vital capacity. Children 4–14 years, adolescents 14–25 years, adults > 25 years.

There were no statistically significant differences in z-scores for FVC, FEV-1, FVC/FEV-1, FEF-25–75, and FEF 75 between SARS-CoV-2 seropositive and seronegative participants (Figure 1 and Supplementary Table 1) in the whole cohort or in the subgroups of children, adolescents and adults. Mean spirometry parameters were within the normal ranges in all subgroups. Patient-reported smoker status, asthma, and pollen allergies were not associated with reduced spirometry parameters.

### Correlation of Spirometry With Clinical Symptoms

**FIGURE 2:**
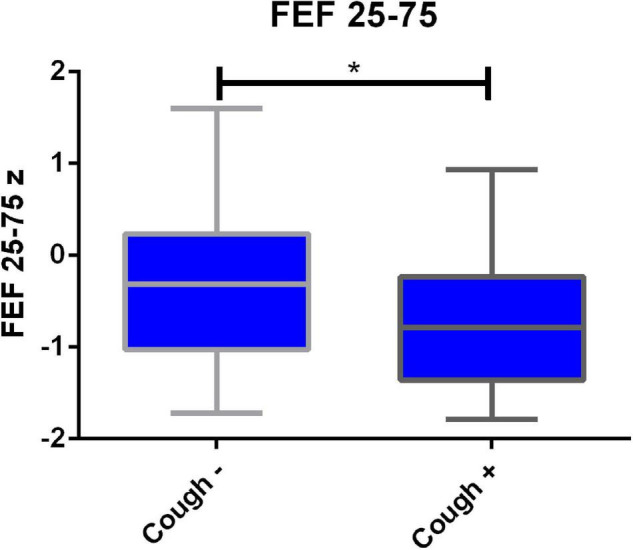
Diminished FEF 25–75 z-score in all seropositive participants with cough during acute SARS-CoV-2 infection 12 months after the infection. Median, quartiles, minimum and maximum values in participants without (*n* = 66) and with cough (*n* = 42) during SARS-CoV-2 infection are visualized. *p < 0.05. FEF25–75z, normalized mean flow between 25% and 75% of the forced vital capacity.

Clinical symptoms (fever, cough, diarrhea, and dysgeusia) during acute infection were correlated with spirometry results. Spearman correlation for the symptom “cough” showed significant correlations for FEV1/FVCz, FEF25–75z and FEF75z (rs respectively: −0.32, −0.36 and −0.32). After adjusting for smoker status, age, and BMI, only cough during acute infection was correlated with lower z-score for FEF25–75 (*p* = 0.03, slope 0.53) (Figure 2 and Table 2).

**TABLE 2:** Results for normalized FEF 25–75 (z-score) measured at T2 for adults that reported symptoms during acute SARS-CoV-2 infection.

	Cough	No cough	*p*-value
Included participants	*n* = 42	*n* = 66	
FEF 25–75z median (IQR)	−1.02 (1.13)	−0.18 (1.26)	0.03

*IQR, interquartile range; n, number of participants.*

### Persistent Symptoms

**FIGURE 3:**
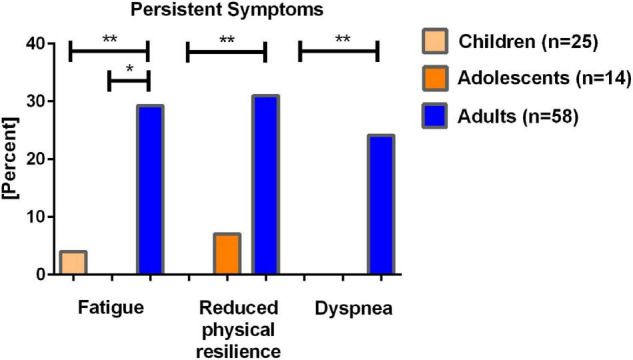
Self-reported persistent clinical symptoms in seropositive children, adolescents and young adults and their parents 12 months after SARS-CoV-2 infection. Data from questionnaires of *n* = 25 children, *n* = 14 adolescents, and *n* = 58 adults. *p < 0.05, **p < 0.001.

Questionnaires regarding persistent symptoms after SARS-CoV-2 infection were available for 58 seropositive adults (93.5% response rate), 14 adolescents (87.5%), and 25 children (89.3%) at T2, respectively. Adults reported more persistent symptoms compared to adolescents and children (Figure 3). Only 4.0% (*n* = 1) of children, and no adolescents reported persistent fatigue since their SARS-CoV-2 infection as did 29.3% (*n* = 17) of adults. Decreased physical resilience after SARS-CoV-2 infection was reported by 7.1% (*n* = 1) of adolescents, and 31.0% (*n* = 18) of adults 12 months after the infection; it was not reported in children. Dyspnea was reported 24.1% (*n* = 14) of adults; it was not reported in adolescents and children.

## Discussion

This study reports spirometry outcomes and persistent subjective symptoms 12 months after mild SARS-CoV-2 infection in children, adolescents and adults. We have found no differences in spirometry parameters between individuals with history of SARS-CoV-2 infection and healthy controls. Only a subgroup of participants who experienced cough during acute infection showed a lower FEF 25–75z compared to those without cough. However, the results still lie within the normal range and are likely not clinically relevant. Additionally, this study found no evidence for a correlation between reported persistent symptoms as fatigue, reduced physical resilience, and dyspnea, and spirometry values.

Pathological pulmonary function tests, especially reduced CO- diffusion capacity, have been reported to persist even after discharge of SARS-CoV-2 infected patients with moderate or severe pulmonary involvement (17, 29–31). Spirometry parameters 2–3 months after discharged have been reported to be reduced but a normalization of all values with the exception of FVC has been demonstrated at 6 months (32–34). Fewer data exist regarding patients with milder course who were not treated as inpatients. One study showed that patients who were still symptomatic 2 months after first symptom onset did show reduced FEV-1 and vital capacity as well as impaired CO-diffusion capacity and elevated airway resistance (20). A Swiss study found no changes in spirometry in milder cases 4 months after SARS-CoV-2 infection (19). Regarding children and adolescents, one study reported no changes in spirometry or CO-diffusion capacity 2 months after SARS-CoV-2 infection in seven children (35) and one pre-print reports no abnormalities in spirometry or multiple breath washout in 73 children and adolescent 2.6 months after infection (36).

The cohort reported here is unique due to its composition of family members of all age groups. Even though direct comparison with other studies is difficult, it is reassuring that 12 months after mild or asymptomatic SARS-CoV-2 (the longest interval compared with the other published studies) no objective changes can be found in spirometry in all three age groups.

Other measures of pulmonary function as body plethysmography (19, 20, 37), impulse oscillometry (38), CO-diffusion capacity (17, 29–31, 37, 39), spiroergometry (40), 6-min-walk-test (19, 41), or multiple-breath-washout (36, 42) have been applied to pulmonary follow-up after SARS-CoV-2 infection with varying results. CO-diffusion capacity, possibly in combination with spiroergometry, currently seems to be the method of choice to monitor patients with persistent symptoms after moderate to severe acute infection, as suggested by professional medical societies and recent guidelines (43, 44).

In this study, correlation of clinical symptoms with spirometry showed only mild reduction in FEF25–75z in adults who had reported cough during acute infection. Some previous studies have reported mild reduction of spirometry parameters (20, 45) in symptomatic patients, others did not (36, 46). All studies show spirometry parameters in the normal ranges—as does the study reported here. Therefore, mild symptomatic SARS-CoV-2 infection does not lead to measurable changes in spirometry in children, adolescents, and adults. Whether cough in our cohort during acute infection is an indication of more pulmonary involvement and therefore more severe disease or that underlying conditions as airway hyperreactivity or asthma, unknown to the patients and therefore not reported, cannot be definitively clarified with this study. All changes in spirometry found in this study are subtle and spirometry results are still in the normal ranges and therefore most likely not of clinical relevance.

In the cohort reported here almost 25% of adults but no children or adolescents reported dyspnea 12 months after SARS-CoV-2 infection and more than 30% of adults but only 7.1% of adolescents and no children reported reduced physical resilience. Prevalence of persisting subjective symptoms after SARS-CoV-2 infection is low in children (47) whereas, depending on the study, around 30% of adults, who had not been hospitalized, reported ongoing symptoms between 3 weeks and 9 months after infection (30, 48–50). Pulmonary symptoms as cough mainly resolved within weeks after infection (16) but dyspnea may persist several months up to 1 year in adults (16, 17). Adolescents, especially teenage girls, seem to suffer more frequently from long-term sequelae (27, 51) but children do not (27, 52). The data reported here are in concordance with the published literature and support the evidence that long-term sequelae after SARS-CoV-2 infection in children are rare. Some authors have suggested a possible link to dysfunctional breathing (for example inducible laryngeal obstruction) after SARS-CoV-2 infection (36), a condition that is well known to occur after other (infectious) triggers (53). Further studies are needed to elucidate this possible pathological link.

The major strength of this study is that it reports clinical and lung function data in multiple age groups (children, adolescents and adults) after the most common clinical scenario (asymptomatic or mild SARS-CoV-2 infection).

Limitations of the study are the single-center design and the lack of longitudinal spirometry data. Additionally, a more detailed evaluation including CO-diffusion capacity and exercise testing would have been desirable. The evaluated subgroups are small, especially the adolescent group. This group does include teenagers and young adults and is quite heterogeneous. It was decided to keep the three age groups as they best represent the structure of participating families. The data on symptoms during the acute phase of SARS-CoV-2 infection as well as on the persistent symptoms on follow-up have to be interpreted with caution. It is based on self-reporting that may be biased.

## Conclusion

Our data show that mild SARS-CoV-2 infection in a household cohort including children, adolescents and adults did not cause persistent, clinically relevant, functional pulmonary abnormalities detectable with spirometry. Self-perceived persistent symptoms as fatigue, reduced physical resilience and dyspnea were more common in adults, less in adolescents and rare in children. The presence of symptoms was not associated with reduced spirometry parameters. More sensitive pulmonary function tests (e.g., spiroergometry, CO-diffusion), would be needed to evaluate subtle pulmonary changes in detail. Despite this reassuring finding, a pulmonary follow-up should be offered to all patients after SARS-CoV-2 infection with persistent respiratory symptoms, since dysfunctional breathing might possibly be the underlying cause in some patients.

## Data Availability Statement

Data are available upon reasonable request from the corresponding author.

## Ethics Statement

The studies involving human participants were reviewed and approved by Ulm University Ethics Committee (Permit No. 152/20). Written informed consent to participate in this study was provided by the participants’ legal guardian/next of kin.

## Author Contributions

SB designed the study, performed the spirometry, analyzed the data, and wrote the first draft of the manuscript. DF performed the spirometry and analyzed the data. MH analyzed the data and prepared the graphs. BM gave critical input to statistical analysis and analyzed the data. MZ coordinated the study and curated data. AH and PF designed online questionnaire and analyzed the data. RE, HR, and MS analyzed the data and gave valuable input to the manuscript. K-MD procured funding and provided key resources. AJ conceived the study, analyzed the data, and performed the spirometry. All authors critically revised the manuscript and agreed to the final version of the manuscript.

## Conflict of Interest

The authors declare that the research was conducted in the absence of any commercial or financial relationships that could be construed as a potential conflict of interest.

## Publisher’s Note

All claims expressed in this article are solely those of the authors and do not necessarily represent those of their affiliated organizations, or those of the publisher, the editors and the reviewers. Any product that may be evaluated in this article, or claim that may be made by its manufacturer, is not guaranteed or endorsed by the publisher.

## Footnotes

1
https://coronavirus.jhu.edu/map.html

2
https://www.project-redcap.org/

3
www.graphpad.com
