# Supplementary material for: Pulmonary Function and Persistent Clinical Symptoms in Children and Their Parents 12 Months After Mild SARS-CoV-2 Infection
Source: Front Pediatr. 2022 Jun 30;10:894331. doi: 10.3389/fped.2022.894331 (PMC9279894; doi:10.3389/fped.2022.894331)
Supplement: Supplementary file 1 [file Data_Sheet_1.PDF]

Supplementary table 1

		Children (median [min;max] mean±SD)	Adolescents (median [min;max] mean±SD)	Adults (median [min;max] mean±SD)	Entire cohort (median [min;max] mean±SD)
FVC z	SARS-CoV-2 positive	-0.45 [-4.49;1.55]<br>-0.73±0.91	-0.69 [-2.42; 1.19]<br>-0.61±0.94	0.53 [-1.36;2.13]<br>0.53±0.80	0.11 [-4.49;2.13]<br>0.03±1.15
	p-value	0.68	0.47	0.85	0.33
	SARS-CoV-2 negative	-0.88 [-1.99;1.68]<br>-0.68±0.91	-0.39 [-2.10;2.24]<br>-0.25±1.35	0.59 [-1.27;2.12]<br>0.56±0.97	-0.17 [-2.10;2.24]<br>-0.06±1.18
FEV1 z	SARS-CoV-2 positive	-0.37 [-3.85;1.90]<br>-0.52±1.52	-0.62 [-3.05;1.55]<br>-0.62±1.15	0.20 [-1.36;2.13]<br>0.12±0.78	0.05 [-3.85;1.90]<br>M=-0.15 SD=1.12
	p-value	0.59	0.44	0.83	0.46
	SARS-CoV-2 negative	-0.55 [-2.37;1.25]<br>-0.63±0.84	-0.38 [-2.42; 2.46]<br>-0.14±1.40	0.24 [-1.59;2.18]<br>0.18±0.90	-0.28 [-2.42;2.46]<br>-0.17±1.07
FVC/FEV1z	SARS-CoV-2 positive	0.69 [-2.46;2.77]<br>0.50±1.35	-0.35 [-1.75;2.31]<br>-0.08±1.21	0.11 [-2.24;3.8]<br>0.09±0.95	0.15 [-2.46;3.81]<br>0.17±1.11
	p-value	0.57	0.23	0.64	0.81
	SARS-CoV-2 negative	0.40 [-2.84;2.32]<br>0.20±1.35	0.35 [-2.95;2.2]<br>0.32±1.24	-0.05 [-2.30;2.18]<br>-0.00±1.03	0.18 [-2.95;2.32]<br>0.15±1.19
FEF25-75 z	SARS-CoV-2 positive	-0.27 [-3.04;2.22]<br>-0.15±1.41	-0.75 [-2.61;1.50]<br>-0.62±1.14	-0.47 [-1.79;2.35]<br>-0.46±0.90	-0.49 [-3.04;2.35]<br>-0.42±1.07
	p-value	0.46	0.13	0.77	0.56
	SARS-CoV-2 negative	-0.39 [-2.88;2.21]<br>-0.47±0.96	0.14 [-2.29;1.29]<br>-0.02±1.00	-0.71 [-2.20;2.23]<br>-0.42±1.20	-0.42 [-2.88;2.23]<br>-0.33±1.08
FEF75 z	SARS-CoV-2 positive	0.47 [-4.45;2.98]<br>0.44±1.90	-0.17 [-2.66;2.98]<br>-0.20±1.57	-0.76 [-1.73;1.62]<br>-0.60±0.70	-0.53 [-4.45;5.95]<br>-0.28±1.31
	p-value	0.13	0.19	0.79	0.37
	SARS-CoV-2 negative	-0.19 [-3.23;3.11]<br>-0.10±1.35	0.32 [-1.05;2.11]<br>0.31±0.84	-0.79 [-1.5;2.2]<br>-0.5±1.00	-0.32 [-3.23;3.11]<br>-0.17±1.13

Supplementary table 1: Spirometry values in children and their parents 12 months after mild SARS-CoV-2 infection. All medians are in the normal range. Normalized values as z-Score (z) are reported. FVC = forced vital capacity, FEV1 = forced expiratory volume in 1 second, FEF25-75 = Mean flow between 25% and 75% of the forced vital capacity, FEF75 = Maximum expiratory flow at 75% expiration of forced vital capacity, SD = standard deviation. n = 108 seropositive participants (n = 53 children (4-14 years), n = 34 adolescents (4-25 years), n = 95 adults (> 25 years)).
